# A quantitative model for cyclin-dependent kinase control of the cell cycle: revisited

**DOI:** 10.1098/rstb.2011.0082

**Published:** 2011-12-27

**Authors:** Frank Uhlmann, Céline Bouchoux, Sandra López-Avilés

**Affiliations:** Chromosome Segregation Laboratory, Cancer Research UK London Research Institute, 44 Lincoln's Inn Fields, London WC2A 3LY, UK

**Keywords:** cell cycle, S phase, mitosis, cyclin-dependent kinase (Cdk), phosphatases, quantitative biology

## Abstract

The eukaryotic cell division cycle encompasses an ordered series of events. Chromosomal DNA is replicated during S phase of the cell cycle before being distributed to daughter cells in mitosis. Both S phase and mitosis in turn consist of an intricately ordered sequence of molecular events. How cell cycle ordering is achieved, to promote healthy cell proliferation and avert insults on genomic integrity, has been a theme of Paul Nurse's research. To explain a key aspect of cell cycle ordering, sequential S phase and mitosis, Stern & Nurse proposed ‘A quantitative model for cdc2 control of S phase and mitosis in fission yeast’. In this model, S phase and mitosis are ordered by their dependence on increasing levels of cyclin-dependent kinase (Cdk) activity. Alternative mechanisms for ordering have been proposed that rely on checkpoint controls or on sequential waves of cyclins with distinct substrate specificities. Here, we review these ideas in the light of experimental evidence that has meanwhile accumulated. Quantitative Cdk control emerges as the basis for cell cycle ordering, fine-tuned by cyclin specificity and checkpoints. We propose a molecular explanation for quantitative Cdk control, based on thresholds imposed by Cdk-counteracting phosphatases, and discuss its implications.

## 1. Introduction

Cell growth and division are the basis for biological life the way we know it. Several chapters in this issue discuss how cell growth and division are regulated so as to fulfil the requirements during birth, development and reproduction of complex multi-cellular organisms, a question of outstanding importance (see the reviews by van Werven & Amon [1], O'Farrell [2] and Kronja & Orr-Weaver [3] in this issue). Here, we focus our attention on the basic molecular machinery that acts within eukaryotic cells to bring about the cell division cycle, the understanding of which was, in many seminal ways, influenced by Paul Nurse's research. While many cellular components approximately double in number during cell growth, before being roughly divided up between daughter cells at cell division, accurate cell cycle control has evolved to guard duplication and segregation of the genome. The genomic DNA is replicated during S phase and then packed into chromosomes for distribution into two newly forming daughter cells in mitosis. The oscillating activity of cyclin-dependent kinases (Cdks) acts as the master regulator for cell cycle progression. Less clear is how these Cdk oscillations are translated into an ordered series of cellular events, which will be the topic of this review.

In simple prokaryotic life forms, S phase and chromosome segregation are thought to be inherently linked, although recent evidence for a sophisticated cellular segregation machinery suggests that unanticipated levels of control may exist [4]. In eukaryotes, it has been argued that the increased genome size made it necessary to separate S phase from mitosis. This is because DNA replication and chromosome condensation, required to compact large chromosomes, might be mutually exclusive [5,6]. The compaction of DNA in a bacterial nucleoid, however, is no less than that in a eukaryotic mitotic chromosome, which renders this explanation insufficient to explain the need to separate S phase and mitosis. In addition to chromosome condensation, cell division in eukaryotes requires the reorganization of many cellular components. The cytoskeleton is reshaped to form a mitotic spindle, instead of defining cell shape and growth zones. In higher eukaryotes, this is helped by centrioles that cease to form the basis for the primary cilium. Intracellular membrane compartments, required for protein sorting and secretion, are disassembled. In many cells, the nuclear envelope, which has set up specialized environments for transcription and translation, breaks down. In a multi-cellular context, cells lose contact with their neighbours. It seems advantageous to confine these unavoidable disruptions to cell physiology during cell division to as short a time window as possible. As S phase typically takes up about one-third of the cell cycle duration in proliferating eukaryotic cells, a shorter dedicated period of mitosis, after DNA replication is complete, will minimize the disruption.

The question of how S phase and mitosis are ordered during the cell cycle gained urgency by the discovery that the same catalytic subunit of Cdk in fission yeast, Cdc2, promotes both entry into S phase as well as subsequent mitosis [7]. The same turned out to be true for the budding yeast Cdk catalytic subunit Cdc28 [8]. Possible solutions for how one enzyme can fulfil two apparently very different roles at different times of the cell cycle were soon put forward [6,9–12]. Among those were the following three, not mutually exclusive, ideas that still make a claim on explaining cell cycle ordering today. (i) Late events are prevented from occurring until completion of earlier events by the action of checkpoints or surveillance mechanisms [9,13]. (ii) Ordering is achieved by different cyclins that associate with the Cdk at different times in the cell cycle [14–16]. (iii) The quantitative increase of Cdk activity during the cell cycle triggers first S phase at a relatively low level, then mitosis as Cdk activity peaks [6,17]. We will consider these three models shortly in more detail, together with relevant evidence that has accumulated since they were first proposed.

The discourse of cell cycle ordering has often focused on sequential S phase and mitosis. On closer inspection, each individual phase of the cell cycle again consists of an intricately ordered series of events. For example, S phase encompasses a temporal programme of early and late replication origin firing [18]. Mitosis in turn is made up of sequential steps that are so characteristic that they carry their own names. Chromosomes are bioriented on the mitotic spindle in metaphase, and once this is achieved a cascade of events is set in motion. Sister chromatids split and segregate to opposite cell poles in anaphase, pulled by the elongating anaphase spindle. After this, chromosomes decondense and the spindle disassembles in telophase, before finally two new daughter cells are pinched off by cytokinesis. All of these latter stages of mitosis have been linked to Cdk downregulation, but how their ordering is achieved is poorly understood.

## 2. Checkpoints or surveillance mechanisms

**Figure 1.**
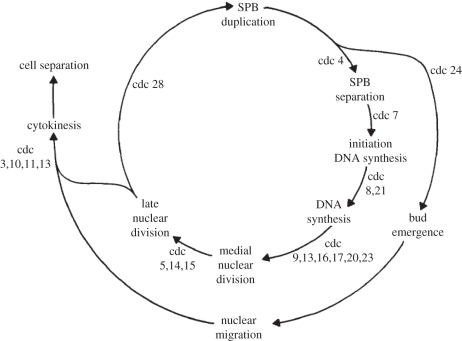
A model in which cell cycle ordering is due to a dependency of events. For example, mitosis (nuclear division) is dependent on the completion of S phase (DNA synthesis). Cells arrest before mitosis if they are deficient in a number of cell division cycle (cdc) genes, including cdc8 and cdc21—encoding thymidylate kinase and thymidylate synthetase required for nucleotide synthesis—and cdc9 and cdc17—encoding DNA ligase I and the catalytic subunit of DNA polymerase *α*, required for DNA replication. This dependency of events suggested that causalities order cell cycle transitions, which later were found to be enforced by checkpoints or surveillance mechanism. Reproduced with permission from Hartwell [20]. Copyright © The Rockefeller University Press.

Two principles of control underlie cell cycle progression. (i) A biochemical oscillator that produces waves of Cdk activity irrespective of the completion of S phase or chromosome segregation [19]. (ii) A wiring diagram of dependent cell cycle events, whose ordering is enforced by checkpoints or surveillance mechanisms (figure 1) [9,20]. According to the latter idea, the ordering of S phase and mitosis depends on a mechanism that prevents Cdk from initiating mitosis until DNA replication is complete.

To understand whether and how such a mechanism operates, we need to revisit what a checkpoint or surveillance mechanism encompasses, as discussed by Nasmyth [5,12]. In everyday language, a checkpoint is a barrier on a street or path that can only be passed if certain conditions are fulfilled. The approach leading up to the checkpoint, in contrast, is not monitored. The checkpoint idea would thus foresee a barrier at the entry into mitosis that can only be passed if all DNA is replicated. According to this idea, the entry point into mitosis, where the checkpoint is located, needs to be defined independently of DNA replication. In this sense, a checkpoint would not solve the problem of defining the time of mitotic entry. However, the term checkpoint is often somewhat misleadingly used to portray what is better described as a surveillance mechanism. According to the idea of a surveillance mechanism, ongoing DNA replication produces a signal, which in turn prevents progression into mitosis until replication is complete and the signal turns off. Does such a surveillance mechanism operate to order S phase and mitosis?

The existence of such a mechanism, aka ‘replication checkpoint’, was inferred from the observation that mutations in DNA polymerases or enzymes that supply deoxynucleotides for DNA synthesis, or the block of deoxynucleotide synthesis by hydroxyurea, prevent cell cycle progression into mitosis [20]. The nature of the signal that is recognized by this surveillance mechanism is still not completely understood. It might be a feature of replication forks that stall in response to polymerase mutation or lack of nucleotides, e.g. persisting regions of single-stranded DNA or of unprocessed RNA primers [21]. Whether replication forks in the process of undisturbed DNA synthesis elicit a similar signal is unclear. Current understanding places the replication surveillance mechanism close to those that recognize DNA breaks or damage [22]. While stalled replication forks, or events that generate DNA damage signals, appear to be commonplace during S phase [23], their stochastic occurrence may not be a reliable measure for ongoing DNA synthesis. In yeasts, components of the DNA damage surveillance mechanisms are not essential for ordered cell cycle progression. Moreover, at least one line of evidence from budding yeast suggests that ongoing DNA replication is indeed invisible to surveillance mechanisms. If, owing to inefficient replication origin licensing, DNA replication in S phase progresses more slowly, mitosis sets in before DNA replication is complete. This results in DNA breaks during chromosome segregation [24]. These considerations suggest that a method different from a surveillance mechanism or checkpoint should exist that orders S phase and mitosis.

Before entirely disregarding the importance of checkpoints for cell cycle ordering, it is worth considering another example, the mitotic checkpoint [25]. This pathway controls the transition from metaphase to anaphase. A single chromosome that is not correctly bioriented on the mitotic spindle delays progression into anaphase, allowing time to correct erroneous and establish correct spindle attachments, which is crucial for correct chromosome segregation. For historical reasons, the mitotic checkpoint is also known as the spindle assembly checkpoint (SAC), in reference to seminal genetic screens in budding yeast that identified many of its molecular components [26,27]. Meanwhile, it is apparent that this checkpoint monitors chromosome biorientation, rather than spindle assembly, most likely by reading out tension between sister kinetochores [28]. Does the mitotic checkpoint qualify as a ‘checkpoint’ and does it order cell cycle progression? Entry into mitosis progresses largely independently of the mitotic checkpoint. Only once cells reach metaphase, the correct attachment of kinetochores decides over cell cycle progression into anaphase. As expected for a checkpoint, kinetochores do not underlie constant surveillance. The monitoring components only assemble at the time when cells enter mitosis. Once cells enter anaphase, the checkpoint has been passed and tension is no longer monitored [29,30]. In this sense, the mitotic checkpoint might well be the only true checkpoint of the cell cycle. Despite its importance for faithful chromosome segregation, its components are not essential in budding yeast [26,27]. The ordering of cell cycle progression thus appears, in principle, checkpoint-independent. In mammalian organisms, in contrast, the mitotic checkpoint is essential. Without it, cells enter anaphase prematurely, leading to chromosome missegregation and cell death [31]. Thus, while not essential for cell cycle ordering, the mitotic checkpoint is part of the mechanism that controls the correct timing of anaphase, at least in mammalian cells.

## 3. Cyclin specificity

**Figure 2.**
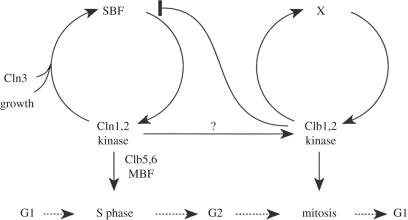
A model for how cyclin specificity orders S phase and mitosis in budding yeast. In this model, G1 and S-phase cyclins (Cln1,2 and Clb5,6) promote S phase, while mitosis is triggered by the mitotic cyclins Clb1,2. G1 cyclins and mitotic cyclins maintain their own activity, respectively, while mitotic cyclins repress G1 cyclins. Reproduced with permission from Amon *et al*. [38]. Copyright © Elsevier.

If not by checkpoints or surveillance mechanisms, how is the ordering of the cell cycle achieved? An important hint came with the discovery of G1 cyclins in budding yeast, Cln1–Cln3, that are required for entry into the cell cycle at the G1–S transition [32,33], followed by the identification of distinct budding yeast cyclins required for mitosis, Clb1–Clb4, more closely related to the previously known metazoan mitotic cyclins [16,34,35]. A third class of cyclins (Clb5 and Clb6) appear at, and are required for, the timely onset of DNA replication in S phase [36,37]. This suggested that different cyclins in the same organism might act at different times to promote sequential cell cycle events. Investigation of the transcriptional control of cyclin expression provided a compelling model for how the cell cycle switches from a G1 stage, dominated by G1 cyclins that maintain their own synthesis and promote expression of S-phase and mitotic cyclins, towards mitosis when mitotic cyclins repress G1 cyclins (figure 2) [38]. Can the thereby generated alternating cyclin waves explain the ordering of the cell cycle? i.e. can only G1 cyclins prime Cdk to phosphorylate proteins that trigger the Start transition and do S-phase cyclins target molecules that initiate DNA replication? Do mitotic cyclins in turn provide specificity for entry into mitosis?

Cyclin subunits are essential adaptors of Cdks that activate the kinase and target it to its substrates. Cyclins are evolutionarily derived from a common ancestor, but G1 cyclins appear sufficiently diverged to make it plausible that they convey a different substrate specificity from S-phase or mitotic cyclins. This idea has been biochemically confirmed in the case of a few G1 cyclin substrates [39]. The understanding of substrate recognition received a boost from crystal structures of the human S-phase cyclin A–Cdk2 complex, bound to the stoichiometric Cdk inhibitor p27 or a fragment of its substrate p107 [40,41]. These structures not only explained the Cdk consensus S/T-P-x-K/R recognition motif. Together with biochemical analyses, they also identified an RxL peptide motif on p27 and Cdk substrates, that is recognized by a hydrophobic patch on the S-phase cyclin [42]. A similar patch is found on G1 cyclins, but is not present in the same shape on mitotic cyclins [43–45]. Following the large-scale identification of Cdk substrates in budding yeast [46], the role of the hydrophobic patch in providing S-phase Cdk substrate recognition was strikingly confirmed [47]. This suggests the RxL motif as a means by which S-phase cyclins recognize specific substrates.

With a rationale for cyclin-specific substrate recognition in hand, is it true that S-phase cyclins are required to trigger S phase? We now know the two crucial Cdk targets whose phosphorylation initiates DNA replication in budding yeast, Sld2 and Sld3 [48,49]. Sld2 is indeed a preferred substrate for Clb5/Cdk, but Sld3 is equally well phosphorylated by the mitotic Clb2/Cdk [47]. A more rigorous test for the importance of S-phase cyclins comes from analyses of cells lacking them. Budding yeast deleted for *clb5*, and its close paralogue *clb6*, are viable but show a delay in S phase [37]. Advanced expression of mitotic Clb2 under control of the Clb5 promoter cannot rescue the delay, which has been taken as an indication that timely DNA replication requires specific substrate phosphorylation by S-phase cyclins [50]. On closer inspection, deletion of the Cdk inhibitory kinase Swe1 in the Clb5 promoter-Clb2 strain was found to restore normal S-phase timing [51]. The reason why Clb2 was slow in triggering S phase therefore turned out to be the stronger negative regulation of Clb2/Cdk, compared with Clb5/Cdk, by Swe1. Once this is corrected, the mitotic cyclin Clb2 is proficient in promoting S phase, and indeed in phosphorylating Sld2 *in vivo*, with normal kinetics [51]. A similar conclusion was reached from experiments replacing *Xenopus* S-phase cyclin A with the mitotic cyclin B in a cell-free extract system that recapitulates cell cycle progression. Cyclin B is normally excluded from interphase nuclei, but removal of its nuclear export signal allowed nuclear accumulation. This change was sufficient for cyclin B to initiate S phase as efficiently as cyclin A would have achieved [52]. Therefore, the ability to trigger S phase is not restricted to S-phase cyclins. Mitotic cyclins are capable of initiating both S phase as well as mitosis, and they do so in the correct order. A more detailed analysis of DNA replication without S-phase cyclins is warranted to discern the possible advantages of the substrate specificity endowed to S-phase cyclins by its RxL recognition motif.

Numerous experiments to delete or replace individual cyclins, or combinations thereof, have been meanwhile performed in various organisms. A few key findings are summarized in table 1 [65]. This shows that the function of most G1 and S-phase cyclins is dispensable for ordered cell cycle progression, or can be made dispensable by compensatory changes in the cell cycle machinery. In contrast, mitotic cyclins are essential. This suggests that the function of G1 and S-phase cyclins can be taken over by mitotic cyclins, but not the other way around. S-phase cyclins cannot substitute for mitotic cyclins even at elevated levels [55,66]. Mitotic cyclins thus appear to be the more generic Cdk activators, with G1 and S-phase cyclins having taken on more specific roles. This is also supported by phylogenetic analyses of cyclins, which place mitotic cyclins at the root of the cyclin tree with S-phase and G1 cyclins being younger derivatives [5]. An example to illustrate this relationship are the budding yeast G1 cyclins. At least one of the three G1 cyclins (Cln1–Cln3) is usually required for cell proliferation [33]. However, they all become dispensable in cells expressing an ectopic source of S-phase cyclins, or if the stoichiometric Cdk inhibitor Sic1 is removed [36,37,53]. Sic1 is part of the mechanism by which mitotic Cdk is downregulated during exit from mitosis. In contrast, Sic1 is a poor inhibitor of G1 cyclins [55]. G1 cyclins are, therefore, destined to overcome Sic1 and turn on the expression of S-phase cyclins during entry into the next cell cycle. In the absence of Sic1, or if S-phase cyclins are expressed from an independent source, G1 cyclins are no longer required. Cells lacking all G1 cyclins and Sic1 are viable, suggesting that G1 cyclin specificity is not essential to achieve ordering of cell cycle progression. Cells lacking Sic1, however, are compromised in maintaining a stable G1 arrest, e.g. in preparation for mating. Thus, cell cycle exit, as part of cellular differentiation, which is promoted by Cdk inhibitors, created a requirement for G1 cyclins that overcome these Cdk inhibitors.

**Table 1.** Cyclin gene deletions and their phenotypes. MEFs, mouse embryonic fibroblasts.

genotype	phenotype	rescue	references
**budding yeast**			
*cln1 cln2 cln3*Δ**	unviable, arrest in G1	*sic1*Δ**, ectopic *CLB5*	[33,36,37,53]
*clb5 clb6*Δ**	viable, delayed S phase	*CLB5*promoter-*CLB2* and *swe1*Δ**	[37,51]
*clb3 clb4 clb1 clb2*Δ**	unviable		[54]
*clb5 clb6 clb3 clb4 clb1*Δ* clb2*^*ts*^*GAL-CLB5*	unviable, arrest in G2		[55]
*clb5 clb6 clb3 clb4 clb1 clb2*Δ* GAL-CLB1*	viable		[56]
**fission yeast**			
*puc1 cig1 cig2*Δ**	viable, delay in G1	*rum1*Δ**	[57]
*cdc13*Δ**	unviable		[58]
**mouse**			
*cycD1*^*−/−*^*D2*^*−/−*^*D3*^*−/−*^	embryos grow but die at mid/late gestation with haematopoietic defects, MEFs are viable		[59]
*cycE1*^*−/−*^*E2*^*−/−*^	death in late embryogenesis, failure of trophoblast giant cell endoreplication, MEFs are viable	wt placenta rescues embryonic development	[60]
*cycA1*^*−/−*^	viable, male infertile		[61]
*cycA2*^*−/−*^	death after day 5.5 p.c., *cycA1*^*−/−*^*A2*^*−/−*^ MEFs are viable		[62,63]
*cycB1*^*−/−*^	death in early embryogenesis		[64]
*cycB2*^*−/−*^	viable and fertile		[64]

In mouse models, numerous cyclins can be deleted with only mild consequences on organismal development and only the major mitotic cyclin B1 is essential for early embryonic cell divisions (table 1). Again, most cyclins appear dispensable for the ordering of cell cycle progression. Instead, some of them have taken on specific roles at particular developmental stages. For example, the S-phase cyclins E1 and E2 together are essential for embryonic development. This is not because of a requirement for cell cycle progression, as cells derived from embryos lacking both cyclins proliferate well in culture. Rather, cyclins E1 and E2 are required to promote the endoreduplication cycles leading to the highly polyploid giant trophoblast nuclei in the placenta. Chimaeric embryos—in which the extra-embryonal cells that give rise to the placenta are wild-type, while the embryo proper lacks both theses cyclins—can be derived. Development is strikingly restored in these cyclin E1- and E2-deficient embryos by the wild-type placenta, with residual cardiovascular abnormalities [60].

In addition to distinct cyclins, vertebrates also encode a number of different catalytic Cdk subunits, including Cdk1, Cdk2, Cdk3, Cdk4 and Cdk6. Depending on their time of activation by the Cdk-activating kinase (CAK), they preferentially associate with the cyclins present at the respective cell cycle stage [67]. Ablation of any of the Cdks, other than the major mitotic Cdk1, has little effect on cell cycle progression and mouse embryonic development. Even simultaneous deletion of Cdk2, Cdk3, Cdk4 and Cdk6, leaving behind only Cdk1, causes only mild delays to cell cycle progression. Cdk1 can substitute for all the other Cdks in these cells and even supports apparently normal mouse embryonic development until mid-gestation. Only after that, a specific requirement of Cdk4 and 6 for haematopoiesis leads to embryonic death owing to anaemia [68]. Taken together, one Cdk subunit is sufficient for setting up orderly cell cycle progression, although individual Cdks have evolved specific functions that they fulfil during certain developmental processes. The molecular nature of these specific functions remains to be elucidated.

## 4. A quantitative model for ordering S phase and mitosis

**Figure 3.**
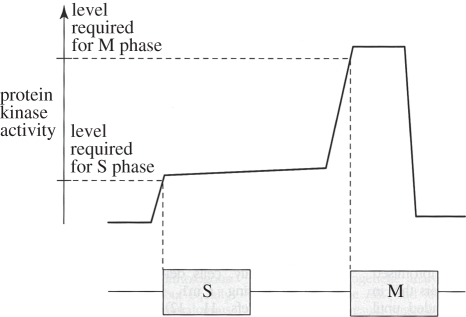
A quantitative model for ordering S phase and mitosis in fission yeast. A single source of Cdk activity (Cdc13/Cdc2) is sufficient for ordering sequential S phase and mitosis. S phase is triggered by an intermediate level of Cdk activity, while mitosis depends on a higher kinase activity level. Reprinted with permission from Stern & Nurse [6]. Copyright © Elsevier.

The fission yeast *Schizosaccharomyces pombe* contains four cyclins: the G1 cyclin Puc1, two S-phase cyclins Cig1 and Cig2 and the mitotic cyclin Cdc13 [58,69–74]. Of those, only Cdc13 is required for cell viability. Cells lacking all three Puc1, Cig1 and Cig2 show a delay in progression through G1 but, apart from this, undergo largely normal cycles of growth and division [75]. The G1 delay is caused by the stoichiometric Cdk inhibitor, Rum1, whose inhibitory effect on Cdk activity is more easily overcome by Puc1 and Cig1. These two cyclins are insensitive to inhibition by Rum1, similar to what is observed for the role of G1 cyclins in budding yeast [57,76]. Cells lacking Rum1 are sterile, again reflecting the role of Cdk inhibitors, and G1 cyclins that overcome them, in cellular differentiation [77]. A difference to budding yeast is that the fission yeast G1 cyclins are not essential even in the presence of Rum1. This might be because Cdc13 synthesis depends to a lesser extent on G1 cyclins in fission yeast as compare with budding yeast. Notably, these observations show that ordered cell cycle progression is achieved in this organism with only a single source of cyclin, Cdc13, associated with a single kinase subunit Cdc2. To explain how cell cycle ordering with a single source of Cdk activity is possible, Stern & Nurse [6] proposed ‘A quantitative model for the cdc2 control of S phase and mitosis in fission yeast’. In this model, ‘different levels of cdc2 activity regulate cell-cycle progression: S phase is initiated when protein kinase activity increases from a very low to a moderate level; maintenance of this moderate level prevents re-initiation of S phase, and a further increase of activity to a high level initiates mitosis’ (figure 3) [6].

The idea of quantitative ordering of the cell cycle appears at first sight simple and powerful. Furthermore, the two distinct levels in Cdk activity required for the sequential occurrence of S phase and mitosis correlate with two major modes of Cdk control. Upon Cdc13 synthesis, a complex between the cyclin and the catalytic subunit Cdc2 forms. This kinase complex gives the signal to initiate S phase; however, at this time, kinase activity is kept attenuated through inhibitory Cdk tyrosine phosphorylation by Wee1. Only once continued Cdc13 synthesis raises the Cdk activity over a certain threshold, a positive feedback loop is engaged that inhibits Wee1 and activates its counteracting phosphatase Cdc25 [78–81]. This second boost lifts Cdk activity high enough to trigger mitosis.

Recent work has probed the quantitative model for Cdk control of S phase and mitosis further and shown that it works even without two distinct levels of Cdk activation [17]. Fission yeast can proliferate with a minimal Cdk control network consisting of a single Cdc13–Cdc2 fusion protein that undergoes cell-cycle-regulated synthesis and destruction, even if the Cdk tyrosine phosphorylation control module has been stripped from the cells. Thus, the increasing concentration of Cdc13–Cdc2 over the course of the cell cycle is sufficient to instruct two sequential cell cycle events, S phase and mitosis. In further support of a purely quantitative control model, regulation of Cdc13–Cdc2 activity in these cells can be achieved at a constant level of the fusion protein by using graded concentrations of a chemical Cdk inhibitor. Cycles of low, intermediate and high Cdk activity imposed by the corresponding inhibitor concentrations are sufficient to drive sequential S phase and mitosis [17]. We do not yet know how this minimal Cdk control network compares to wild-type cells in its ability to respond to environmental challenges and changes. Clearly, in principle, the ordering of cell cycle progression can be achieved with a single oscillating source of Cdk activity. This finding can rationalize the surprising resilience also of other organisms to the deletion of parts of their Cdk control networks. Despite the presence of checkpoints, surveillance mechanisms and multiple cyclins over long evolutionary time spans, the main mitotic cyclin–Cdk complex has retained an astonishing ability to orchestrate most (if not all) essential aspects of cell cycle progression.

## 5. A kinase/phosphatase ratio model for ordering cell cycle progression

The immediate question that the quantitative model for Cdk control raises is how do increasing levels of Cdk activity order sequential cell cycle events? The tacit assumption is that a low level of Cdk activity, or a small concentration of active Cdk complexes, is able to phosphorylate those Cdk substrates that will bring about S phase, but not those whose phosphorylation will promote mitosis. Higher activity levels or a higher concentration of the active kinase are required to phosphorylate the latter substrates. What could be the molecular basis for this ordering? Does the Cdk simply have a higher affinity for its S-phase targets, and could that explain ordered phosphorylation timing? In a survey of *in vitro* phosphorylation of budding yeast Cdk substrates by the major mitotic Clb2/Cdk, a wide range of efficiencies was recorded. These spanned four orders of magnitude, depending on the substrate. However, no obvious correlation emerged between the efficiency of substrate phosphorylation and its expected timing in the cell cycle. Proteins that were phosphorylated by Clb2/Cdk with the highest efficiencies were functionally linked to either DNA replication or to mitosis [46,47]. This suggests that differential catalytic efficiencies of the Cdk for its respective targets are unlikely to be a sufficient explanation for substrate ordering. Furthermore, a greater catalytic efficiency would give early S-phase substrates only a small competitive advantage. With substrate turnover in seconds and micromolar intracellular cyclin concentrations [47,82], even less efficient substrates would become phosphorylated shortly after the efficient substrates. Deferral of phosphorylation until mitosis would be difficult to achieve. Control by surveillance mechanisms that prevent mitotic entry would also be hard to enforce.

Another example where ordering of sequential cell cycle events has been studied is mitotic exit. Cdk activity is downregulated as mitotic cyclins are targeted for destruction by the anaphase-promoting complex (APC). Cyclin recognition by the APC requires a destruction box, and its mutation or deletion allows expression of cyclins that are resistant to degradation [83]. When non-degradable cyclin B1 is expressed in human-cultured cells, exit from mitosis is affected at various steps in a dose-dependent manner. High levels of non-degradable cyclin B1 interfere with anaphase spindle elongation, while lower levels allow anaphase to occur, but block cells in telophase before chromosome decondensation and cytokinesis [84]. Similar observations have been made in budding yeast [85]. Again, quantitative changes in Cdk activity appear to control sequential events, this time in the reverse order of Cdk inactivation. A small reduction of Cdk activity promotes early anaphase events, while greater reduction is required for later events, and ultimately cytokinesis, to occur. While our molecular understanding of mitotic exit events is still scarce, sequential dephosphorylation of mitotic Cdk targets is thought to be the driving force, a few examples of which have been characterized [86–91]. The requirement for protein dephosphorylation during mitotic exit is underscored by the finding in budding yeast that the Cdk counteracting phosphatase Cdc14 is required for execution of many, if not most, mitotic exit events [92,93].

We will now argue that the realization that a Cdk counteracting phosphatase controls the substrate phosphorylation status, as much as the Cdk itself, holds the key to understanding quantitative models of cell cycle ordering. For this, let us consider how sequential Cdk substrate dephosphorylation is achieved during mitotic exit. In budding yeast, the Cdc14 phosphatase is activated by two complementary pathways, Cdc14 early anaphase release (FEAR) and the mitotic exit network (MEN), with the former acting earlier during mitotic exit than the latter [92,93]. One suggestion has, therefore, been that FEAR-activated Cdc14 targets the substrates that are dephosphorylated early during mitotic exit, while MEN-activated Cdc14 dephosphorylates later substrates [94]. Considering that both pathways activate Cdc14 in a similar manner (by releasing it from its inhibitor Net1), it is not clear how FEAR and MEN could make such a qualitative distinction. Instead, the two pathways gradually release Cdc14 from inhibition, the FEAR pathway initially making use of high mitotic Cdk activity, handing over to the MEN pathway as Cdk activity declines [95–97]. In this scenario, cells experience increasing Cdc14 phosphatase activity, while over the same period Cdk activity decreases. The defining parameter that, therefore, changes over the course of mitotic exit is the ratio between the activities of Cdk and of its counteracting phosphatase Cdc14.

**Figure 4.**
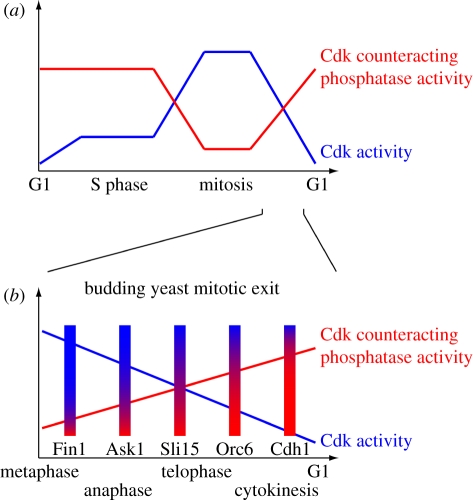
A kinase/phosphatase ratio model for ordering cell cycle progression. (*a*) Sequential phosphorylation or dephosphorylation events are the consequence of substrates responding to distinct thresholds of the changing Cdk to Cdk-counteracting phosphatase ratio. S-phase substrates are efficiently phosphorylated by low levels of Cdk even in the presence of Cdk-counteracting phosphatases. Phosphorylation of mitotic substrates awaits higher Cdk activity levels and phosphatase downregulation. Note that little is still known about the regulation of Cdk counteracting phosphatases. The depicted graph is hypothetical, inspired by what is known about budding yeast Cdc14, fission yeast Clp1 and vertebrate PP2A-B55*δ* [92,99,100]. (*b*) An example of ordered Cdk substrate dephosphorylation during budding yeast mitotic exit. Biochemical evidence shows that the Cdk-counteracting phosphatase Cdc14 targets early dephosphorylated substrates, e.g. Fin1, Ask1 and Sli15, with greater catalytic efficiency and causes their dephosphorylation even in the presence of persisting Cdk activity. Late substrates, involved in spindle disassembly and return of the cell cycle to G1, e.g. Orc6 and Cdh1, are dephosphorylated with lower catalytic efficiency, awaiting a greater Cdc14-to-Cdk ratio [101].

This ratio becomes important if we consider that at any time a Cdk substrate is subjected to both phosphorylation by the Cdk and dephosphorylation by the counteracting phosphatase(s). To maintain the phosphorylated state, continued Cdk activity is required, as has been demonstrated for numerous Cdk substrates [46,98]. Similarly, substrate dephosphorylation by Cdc14 will require not only phosphate removal, but also maintenance of the dephosphorylated state against rephosphorylation by the Cdk. The phosphorylation status of a protein is thus determined by the ratio of the relative rates of phosphorylation and dephosphorylation. As this ratio changes over the course of mitotic exit, substrates shift to their dephosphorylated state. Each substrate will respond at an individual threshold, and therefore timing, depending on its respective efficiencies as a substrate for the Cdk and the phosphatase(s) (figure 4). In a concrete example, budding yeast Fin1 must be dephosphorylated in early anaphase to promote stable anaphase spindle elongation. Fin1 is dephosphorylated by small concentrations of Cdc14 *in vivo* and *in vitro* even while Cdk activity is still present. In contrast, Orc6 remains fully phosphorylated under the same conditions and is dephosphorylated only when Cdc14 reaches higher levels and Cdk activity drops [101]. Orc6 dephosphorylation is part of the mechanism that re-licenses DNA replication origins. To avoid the danger of over-replication, this should not occur before Cdk activity has sufficiently dropped. This differential dephosphorylation timing is explained by the markedly higher catalytic efficiency of Cdc14 for Fin1 when compared with Orc6, while both substrates are equally well phosphorylated by Clb2/Cdk [101].

If we put these considerations back into the context of ordering S phase and mitosis, we suggest that also here a Cdk counteracting phosphatase is crucial to establish thresholds for Cdk phosphorylation of S phase and mitotic substrates, respectively. Such thresholds allow the timing of substrate phosphorylation to be linked to the quantitative increase in Cdk activity during the cell cycle (figure 4). They also have the potential to finetune the phosphorylation timing of individual substrates within S phase and mitosis. The ratio of Cdk phosphorylation versus dephosphorylation efficiency will be greatest for early S-phase substrates. In contrast, substrates phosphorylated late during mitotic entry will be poor substrates for the Cdk compared with the counteracting phosphatase(s). Hints as to the possible identity of the Cdk counteracting phosphatase(s) that help to order S phase and mitosis exist. Mitotic entry in fission yeast is advanced following inactivation of the fission yeast Cdc14 orthologue Clp1 or mutation of the PP2A subunit Ppa2, suggesting that these phosphatases counteract Cdk at this time. The interpretation of these results is somewhat confounded by the impact of these phosphatases also on Cdc25-dependent Cdk activation [102,103]. Recent observations with *Xenopus* cell-free extracts support a role for PP2A, specifically for its PP2A-B55*δ* isoform, as Cdk counteracting phosphatase during mitotic entry in vertebrates. Again, a clear distinction between whether the phosphatase counteracts Cdk substrate phosphorylation or counteracts Cdk activation remains difficult [99,104]. The identity of a Cdk counteracting phosphatase that acts during budding yeast interphase remains to be confirmed.

While the Cdk counteracting phosphatase(s) that set the thresholds for ordering S phase and mitosis thus remain to be verified, several features of the kinase/phosphatase threshold model can already be inferred. (i) A substrate's phosphorylation timing depends on the relative efficiencies of Cdk phosphorylation versus dephosphorylation rather than the absolute Cdk efficiency. This is consistent with the observed broad spread of efficiencies with which Cdk phosphorylates both S phase or mitotic substrates [46]. Individual substrates may be more or less readily accessible to modifications, which might equally affect phosphorylation and dephosphorylation rates. The phosphorylation timing would then be determined by subtle substrate features that finetune the relative catalytic efficiencies of the kinase and phosphatase. (ii) There will be periods in S phase, and during mitotic exit, when the phosphorylation status of certain proteins is maintained by repeated cycles of phosphorylation and dephosphorylation, which may seem wasteful. Every phosphorylation and dephosphorylation cycle of a phosphorylation site will consume 1 ATP. We know little about the rate at which phosphates are turned over *in vivo*, which will vary depending on the catalytic efficiencies of kinase and phosphatase. If we extrapolate from the examples of Kar9 and Slk19, proteins that are phosphorylated by Cdk with intermediate to good efficiency and lose their phosphorylation after Cdk inhibition in about 10 min [47,98], the total expense for this mode of regulation would typically amount to tens of ATP molecules per protein per cell cycle. The expense for this regulation is, therefore, very small compared with the total amount of energy required to synthesize the protein. (iii) The threshold model offers an explanation of how the G2–M transition is controlled by surveillance mechanisms that restrain Cdk activity by inhibiting Cdc25-mediated tyrosine dephosphorylation, which is hard to explain with models that rely on differential efficiencies of forward Cdk substrate phosphorylation. If Cdc25 is inhibited, cells contain an intermediate level of Cdk activity, which will create a new steady-state of Cdk and counteracting phosphatase activities that results in a stable equilibrium in which S-phase, but not mitotic, targets are phosphorylated.

## 6. Conclusions and implications

A quantitative mechanism, based on kinase/phosphatase thresholds, appears to form the basis of ordered cell cycle progression. We suggest that the changing ratio of Cdk and its counteracting phosphatase(s) is read out by individual substrates that respond by changing the phosphorylation status at their respective thresholds, both during the progression through G1, S phase and mitosis, as well as during the sequential steps of exit from mitosis and return to G1. This model can explain how ordered cell cycle progression is possible even with only one cyclin. The mitotic cyclin is the essential and generic Cdk activator, able to control almost any part of the cell cycle. Specific G1 and S-phase cyclins have taken on more specialized roles. These may have less to do with an exclusive range of substrate specificities, but rather with their ability to overcome specific modes of inhibition by stoichiometric Cdk inhibitors in G1, or inhibitory Cdk phosphorylation in G2 phase. These levels of control permit additional crosstalk of the cell cycle with extracellular signals during development and differentiation or in response to intracellular surveillance mechanisms, both of which make use of these inhibitors.

A largely quantitative model of cell cycle control poses challenges and offers explanations. A key challenge is to understand how the timings of mitotic entry and exit, which are ultimately dictated by the rates of cyclin synthesis and destruction, are adjusted to the needs of their associated physiological events. If it defines the timing of mitotic entry, and ongoing replication has little influence on it, the rate of cyclin synthesis must be accurately set such that mitotic entry occurs once DNA replication is complete. Clearly, if Cdk activity raises too quickly, the ordering of S phase and mitosis is compromised. *Xenopus* extracts supplemented with too much nuclear targeted cyclin B initiate S phase, but DNA replication comes to a premature halt as extracts enter mitosis too early [52]. In fission yeast, an accelerated rise in Cdk activity also leads to mitotic entry with incompletely replicated chromosomes and consequent mitotic catastrophe [17]. The principles that produce the correct rate of Cdk activation merit further investigation. The need for completion of DNA replication within a certain time window emphasizes the importance of the ‘random gap problem’. This is caused by the stochastic firing of DNA replication origins, which makes it unavoidable that sporadically replication gaps remain by the time cells enter mitosis. Models of how this random gap problem might be solved have been put forward, and they remain an important area of further research [18].

On the other hand, a quantitative view of the cell cycle can help us to solve apparent puzzles, produced if genetic wiring diagrams are interpreted as strictly causal relationships with all or nothing responses. A frequently raised issue about mitotic entry control is the following apparent paradox: if Cdk activity is inhibited by Cdk tyrosine phosphorylation, but at the same time Cdk activity is required to trigger the positive feedback loop for its activation (by Wee1 and Cdc25 phosphorylation), how will Cdk ever be able to come out of its inhibited state? In a quantitative view of cell cycle regulation, this paradox resolves naturally. There is no ‘zero’ in dynamic control networks; so even tyrosine phosphorylated Cdk displays a basal level of activity, and even unphosphorylated Cdc25 is not entirely inactive. The resulting basal rate of Cdk activity, however, is still limited by the availability of cyclins. Thus, cyclin synthesis will cause a slow increase in Cdk activity, initially sufficient only to trigger S phase. Eventually, as cyclin levels continue to rise, the threshold for engaging the feedback loops will be reached, triggering the rapid rise in Cdk activity that drives mitotic entry [105].

A final remark refers to another enigmatic concept in cell cycle regulation, that of cell size control. It is poorly understood how cells maintain a constant size during repeated rounds of division, and are able to adapt to a new larger or smaller size while following a developmental programme [106]. A common thought is that certain cell size checkpoints or surveillance mechanisms exist that monitor size and feed back to the cell cycle machinery to adjust cycle progression to cell growth. Some of Paul Nurse's own early work in Murdoch Mitchison's laboratory [107] was dedicated to the study of mutant fission yeast strains in which the coordination between cell growth and division is disrupted. A mutation that caused cells to divide at about half their normal size turned out to define a key generic cell cycle regulator, the Cdk inhibitory kinase Wee1 [78,108]. Meanwhile, a mechanism that is geared to measure fission yeast cell size has been described, but its impact on cell size homeostasis and cell cycle progression is small and may be specific to this organism [109,110]. Screens to identify cell size regulators in budding yeast have again yielded generic regulators of the cell cycle as well as of ribosome biogenesis [111]. Why has no specific cell size surveillance mechanism or checkpoint been uncovered? If one considers the cell cycle control network as a quantitative system, put into motion by cyclin synthesis and destruction, then no specialized cell size control pathway might be required. A defined cell size is likely an emergent property of this network. The intracellular concentration of cyclin required to trigger cell cycle transitions is reached in the process of its synthesis by transcription and translation. Cyclin synthesis in turn is inherently coupled to that of all other cellular components, whose expression levels relative to each other are remarkably constant over a large range of cell sizes in fission yeast [112]. Unavoidably, therefore, cells will have reached a certain reproducible size at the time cyclin levels reach the thresholds to trigger S phase or mitosis. In this scenario, mutations in cell cycle regulators will change the cell size at particular cell cycle transitions by changing the respective required cyclin concentration. Changes to the protein expression machinery in turn might impact on cell size by changing the relative rates of cyclin synthesis compared with that of other cellular components. This corresponds to the spectrum of mutations so far discovered in the search for cell size regulators. In budding yeast, nutrient availability regulates G1 cyclin expression rates, and thereby changes the cell size at which the required cyclin concentration for S phase is reached [113]. A model has also been proposed on how a quantitative G1 cyclin threshold for entry into S phase might function [114]. In how far this simple, quantitative view of cell size control holds up to scrutiny remains to be seen.

## References

[1] van Werven F. J. Amon A. 2011 Regulation of entry into gametogenesis. Phil. Trans. R. Soc. B. 366, 3521–3531 10.1098/rstb.2011.0081 (doi:10.1098/rstb.2011.0081) PMC3203461 22084379

[2] O'Farrell P. H. 2011 Quiescence: early evolutionary origins and universality do not imply uniformity. Phil. Trans. R. Soc. B. 366, 3498–3507 10.1098/rstb.2011.0079 (doi:10.1098/rstb.2011.0079) PMC3203459 22084377

[3] Kronja I. Orr-Weaver T. L. 2011 Translational regulation of the cell cycle: when, where, how and why? Phil. Trans. R. Soc. B. 366, 3638–3652 10.1098/rstb.2011.0084 (doi:10.1098/rstb.2011.0084) PMC3203463 22084390

[4] Ptacin J. L. Lee S. F. Garner E. C. Toro E. Eckart M. Comolli L. R. Moerner W. E. Shapiro L. 2010 A spindle-like apparatus guides bacterial chromosome segregation. Nat. Cell Biol. 12, 791–798 10.1038/ncb2083 (doi:10.1038/ncb2083) 20657594 PMC3205914

[5] Nasmyth K. 1995 Evolution of the cell cycle. Phil. Trans. R Soc. Lond. B 349, 271–281 10.1098/rstb.1995.0113 (doi:10.1098/rstb.1995.0113) 8577838

[6] Stern B. Nurse P. 1996 A quantitative model for the cdc2 control of S phase and mitosis in fission yeast. Trends Genet. 12, 345–350 8855663

[7] Nurse P. Bissett Y. 1981 Gene requirement in G_1_ for commitment to cell cycle and in G_2_ for control of mitosis in fission yeast. Nature 292, 558–560 10.1038/292558a0 (doi:10.1038/292558a0) 7254352

[8] Piggott J. R. Rai R. Carter B. L. A. 1982 A bifunctional gene product involved in two phases of the cell cycle. Nature 298, 391–393 10.1038/298391a0 (doi:10.1038/298391a0) 7045699

[9] Hartwell L. H. Weinert T. A. 1989 Checkpoints: controls that ensure the order of cell cycle events. Science 246, 629–634 10.1126/science.2683079 (doi:10.1126/science.2683079) 2683079

[10] Murray A. W. Kirschner M. W. 1989 Dominoes and clocks: the union of two views of the cell cycle. Science 246, 614–621 10.1126/science.2683077 (doi:10.1126/science.2683077) 2683077

[11] Hayles J. Fisher D. Woollard A. Nurse P. 1994 Temporal order of S phase and mitosis in fission yeast is determined by the state of the p34^*cdc2*^–mitotic B cyclin complex. Cell 78, 813–822 10.1016/S0092-8674(94)90542-8 (doi:10.1016/S0092-8674(94)90542-8) 8087848

[12] Nasmyth K. 1995 How do cells control the timing of DNA replication and mitosis? Harvey Lect. 88, 141–171 1365873

[13] Weinert T. A. Hartwell L. H. 1988 The *RAD9* gene controls the cell cycle response to DNA damage in *Saccharomyces cerevisiae*. Science 241, 317–322 10.1126/science.3291120 (doi:10.1126/science.3291120) 3291120

[14] Enoch T. Nurse P. 1990 Mutation of fission yeast cell cycle control genes abolishes the dependence of mitosis on DNA replication. Cell 60, 665–673 10.1016/0092-8674(90)90669-6 (doi:10.1016/0092-8674(90)90669-6) 2406029

[15] Minshull J. Golsteyn R. Hill C. S. Hunt T. 1990 The A- and B-type cyclin associated cdc2 kinase in *Xenopus* turn on and off at different times in the cycle. EMBO J. 9, 2865–2875 2143983 10.1002/j.1460-2075.1990.tb07476.x PMC551999

[16] Surana U. Robitsch H. Price C. Schuster T. Fitch I. Futcher A. B. Nasmyth K. 1991 The role of CDC28 and cyclins during mitosis in the budding yeast *S. cerevisiae*. Cell 65, 145–161 10.1016/0092-8674(91)90416-V (doi:10.1016/0092-8674(91)90416-V) 1849457

[17] Coudreuse D. Nurse P. 2010 Driving the cell cycle with a minimal CDK control network. Nature 468, 1074–1079 10.1038/nature09543 (doi:10.1038/nature09543) 21179163

[18] Rhind N. 2006 DNA replication timing: random thoughts about origin firing. Nat. Cell Biol. 8, 1313–1316 10.1038/ncb1206-1313 (doi:10.1038/ncb1206-1313) 17139278 PMC2861037

[19] Gerhart J. Wu M. Kirschner M. 1984 Cell cycle dynamics of an M-phase-specific cytoplasmic factor in *Xenopus laevis* oocytes and eggs. J. Cell Biol. 98, 1247–1255 10.1083/jcb.98.4.1247 (doi:10.1083/jcb.98.4.1247) 6425302 PMC2113233

[20] Hartwell L. H. 1978 Cell division from a genetic perspective. J. Cell Biol. 77, 627–637 10.1083/jcb.77.3.627 (doi:10.1083/jcb.77.3.627) 355261 PMC2110141

[21] Michael W. M. Ott R. Fanning E. Newport J. 2000 Activation of the DNA replication checkpoint through RNA synthesis by primase. Science 289, 2133–2137 10.1126/science.289.5487.2133 (doi:10.1126/science.289.5487.2133) 11000117

[22] Bartek J. Lukas C. Lukas J. 2004 Checking on DNA damage in S phase. Nat. Rev. Mol. Cell Biol. 5, 793–804 10.1038/nrm1493 (doi:10.1038/nrm1493) 15459660

[23] Lisby M. Rothstein R. Mortensen U. H. 2001 Rad52 forms DNA repair and recombination centers during S phase. Proc. Natl Acad. Sci. USA 98, 8276–8282 10.1073/pnas.121006298 (doi:10.1073/pnas.121006298) 11459964 PMC37432

[24] Lengronne A. Schwob E. 2002 The yeast CDK inhibitor Sic1 prevents genomic instability by promoting replication origin licensing in late G_1_. Mol. Cell 9, 1067–1078 10.1016/S1097-2765(02)00513-0 (doi:10.1016/S1097-2765(02)00513-0) 12049742

[25] Santaguida S. Musacchio A. 2009 The life and miracles of kinetochores. EMBO J. 28, 2511–2531 10.1038/emboj.2009.173 (doi:10.1038/emboj.2009.173) 19629042 PMC2722247

[26] Li R. Murray A. W. 1991 Feedback control of mitosis in budding yeast. Cell 66, 519–531 10.1016/0092-8674(81)90015-5 (doi:10.1016/0092-8674(81)90015-5) 1651172

[27] Hoyt M. A. Totis L. Roberts B. T. 1991 *S. cerevisiae* genes required for cell cycle arrest in response to loss of microtubule function. Cell 66, 507–517 10.1016/0092-8674(81)90014-3 (doi:10.1016/0092-8674(81)90014-3) 1651171

[28] Liu D. Vader G. Vromans M. J. M. Lampson M. A. Lens S. M. A. 2009 Sensing chromosome bi-orientation by spatial separation of aurora B kinase from kinetochore substrates. Science 323, 1350–1353 10.1126/science.1167000 (doi:10.1126/science.1167000) 19150808 PMC2713345

[29] Palframan W. J. Meehl J. B. Jaspersen S. L. Winey M. Murray A. W. 2006 Anaphase inactivation of the spindle checkpoint. Science 313, 680–684 10.1126/science.1127205 (doi:10.1126/science.1127205) 16825537

[30] Mirchenko L. Uhlmann F. 2010 Sli15^INCENP^ dephosphorylation prevents mitotic checkpoint reengagement due to loss of tension at anaphase onset. Curr. Biol. 20, 1396–1401 10.1016/j.cub.2010.06.023 (doi:10.1016/j.cub.2010.06.023) 20619650 PMC2964898

[31] Dobles M. Liberal V. Scott M. L. Benezra R. Sorger P. K. 2000 Chromosome missegregation and apoptosis in mice lacking the mitotic checkpoint protein Mad2. Cell 101, 635–645 10.1016/S0092-8674(00)80875-2 (doi:10.1016/S0092-8674(00)80875-2) 10892650

[32] Nash R. Tokiwa G. Anand S. Erickson K. Futcher A. B. 1988 The *WHI1*^+^ gene of *Saccharomyces cerevisiae* tethers cell division to cell size and is a cyclin homolog. EMBO J. 7, 4335–4346 2907481 10.1002/j.1460-2075.1988.tb03332.x PMC455150

[33] Richardson H. E. Wittenberg C. Cross F. Reed S. I. 1989 An essential G1 function for cyclin-like proteins in yeast. Cell 59, 1127–1133 10.1016/0092-8674(89)90768-X (doi:10.1016/0092-8674(89)90768-X) 2574633

[34] Minshull J. Blow J. J. Hunt T. 1989 Translation of cyclin mRNA is necessary for extracts of activated *Xenopus* eggs to enter mitosis. Cell 56, 947–956 10.1016/0092-8674(89)90628-4 (doi:10.1016/0092-8674(89)90628-4) 2564315

[35] Ghiara J. B. Richardson H. E. Sugimoto K. Henze M. Lew D. J. Wittenberg C. Reed S. I. 1991 A cyclin B homolog in *S. cerevisiae*: chronic activation of the Cdc28 protein kinase by cyclin prevents exit from mitosis. Cell 65, 163–174 10.1016/0092-8674(91)90417-W (doi:10.1016/0092-8674(91)90417-W) 1849458

[36] Epstein C. B. Cross F. 1992 *CLB5*: a novel B cyclin from budding yeast with a role in S phase. Genes Dev. 6, 1695–1706 10.1101/gad.6.9.1695 (doi:10.1101/gad.6.9.1695) 1387626

[37] Schwob E. Nasmyth K. 1993 CLB5 and CLB6, a new pair of B cyclins involved in DNA replication in *Saccharomyces cerevisiae*. Genes. Dev. 7, 1160–1175 10.1101/gad.7.7a.1160 (doi:10.1101/gad.7.7a.1160) 8319908

[38] Amon A. Tyers M. Futcher B. Nasmyth K. 1993 Mechanisms that help the yeast cell cycle clock tick: G2 cyclins transcriptionally activate G2 cyclins and repress G1 cyclins. Cell 74, 993–1007 10.1016/0092-8674(93)90722-3 (doi:10.1016/0092-8674(93)90722-3) 8402888

[39] Kitagawa M. 1996 The consensus motif for phosphorylation by cyclin D1-Cdk4 is different from that for phosphorylation by cyclin A/E-Cdk2. EMBO J. 15, 7060–7069 9003781 PMC452531

[40] Russo A. A. Jeffrey P. D. Patten A. K. Massague J. Pavletich N. P. 1996 Crystal structure of the p27^Kip1^ cyclin-dependent-kinase inhibitor bound to the cyclin A-Cdk2 complex. Nature 382, 325–331 10.1038/382325a0 (doi:10.1038/382325a0) 8684460

[41] Brown N. R. Noble M. E. M. Endicott J. A. Johnson L. N. 1999 The structural basis for specificity of substrate and recruitment peptides for cyclin-dependent kinases. Nat. Cell Biol. 1, 438–443 10.1038/15674 (doi:10.1038/15674) 10559988

[42] Adams P. D. Sellers W. R. Sharma S. K. Wu A. D. Nalin C. M. Kaelin J. W. G. 1996 Identification of a cyclin-cdk2 recognition motif present in substrates and p21-like cyclin-dependent kinase inhibitors. Mol. Cell. Biol. 16, 6623–6633 8943316 10.1128/mcb.16.12.6623 PMC231664

[43] Petri E. T. Errico A. Escobedo L. Hunt T. Basavappa R. 2007 The crystal structure of human cyclin B. Cell Cycle 6, 1342–1349 10.4161/cc.6.11.4297 (doi:10.4161/cc.6.11.4297) 17495533

[44] Day P. J. 2009 Crystal structure of human CDK4 in complex with a D-type cyclin. Proc. Natl Acad. Sci. USA 106, 4166–4170 10.1073/pnas.0809645106 (doi:10.1073/pnas.0809645106) 19237565 PMC2657441

[45] Takaki T. Echalier A. Brown N. R. Hunt T. Endicott J. A. Noble M. E. M. 2009 The structure of CDK4/cyclin D3 has implications for models of CDK activation. Proc. Natl Acad. Sci. USA 106, 4171–4176 10.1073/pnas.0809674106 (doi:10.1073/pnas.0809674106) 19237555 PMC2657433

[46] Ubersax J. A. Woodbury E. L. Quang P. N. Paraz M. Blethrow J. D. Shah K. Shokat K. M. Morgan D. O. 2003 Targets of the cyclin-dependent kinase Cdk1. Nature 425, 859–864 10.1038/nature02062 (doi:10.1038/nature02062) 14574415

[47] Loog M. Morgan D. O. 2005 Cyclin specificity in the phosphorylation of cyclin-dependent kinase substrates. Nature 434, 104–108 10.1038/nature03329 (doi:10.1038/nature03329) 15744308

[48] Tanaka S. Umemori T. Hirai K. Muramatsu S. Kamimura Y. Araki H. 2007 CDK-dependent phosphorylation of Sld2 and Sld3 initiates DNA replication in budding yeast. Nature 445, 328–332 10.1038/nature05465 (doi:10.1038/nature05465) 17167415

[49] Zegerman P. Diffley J. F. X. 2007 Phosphorylation of Sld2 and Sld3 by cyclin-dependent kinases promotes DNA replication in budding yeast. Nature 445, 281–285 10.1038/nature05432 (doi:10.1038/nature05432) 17167417

[50] Cross F. R. Yuste-Rojas M. Gray S. Jacobson M. D. 1999 Specialization and targeting of B-type cyclins. Mol. Cell 4, 11–19 10.1016/S1097-2765(00)80183-5 (doi:10.1016/S1097-2765(00)80183-5) 10445023

[51] Hu F. Aparicio O. M. 2005 Swe1 regulation and transcriptional control restrict the activity of mitotic cyclins toward replication proteins in *Saccharomyces cerevisiae*. Proc. Natl Acad. Sci. USA 102, 8910–8915 10.1073/pnas.0406987102 (doi:10.1073/pnas.0406987102) 15956196 PMC1157011

[52] Moore J. D. Kirk J. A. Hunt T. 2003 Unmasking the S-phase-promoting potential of cyclin B1. Science 300, 987–990 10.1126/science.1081418 (doi:10.1126/science.1081418) 12738867

[53] Tyers M. 1996 The cyclin-dependent kinase inhibitor p40^*SIC1*^ imposes the requirement for Cln G1 cyclin function at Start. Proc. Natl Acad. Sci. USA 93, 7772–7776 10.1073/pnas.93.15.7772 (doi:10.1073/pnas.93.15.7772) 8755551 PMC38823

[54] Fitch I. T. Dahmann C. Surana U. Amon A. Nasmyth K. Goetsch L. Byers B. Futcher B. 1992 Characterization of four B-type cyclin genes of the budding yeast *Saccharomyces cerevisiae*. Mol. Biol. Cell 3, 805–818 1387566 10.1091/mbc.3.7.805 PMC275636

[55] Schwob E. Böhm T. Mendenhall M. D. Nasmyth K. 1994 The B-type cyclin kinase inhibitor p40^*SIC1*^ controls the G1 to S transition in *S. cerevisiae*. Cell 79, 233–244 10.1016/0092-8674(94)90193-7 (doi:10.1016/0092-8674(94)90193-7) 7954792

[56] Haase S. B. Reed S. I. 1999 Evidence that a free-running oscillator drives G1 events in the budding yeast cell cycle. Nature 401, 394–397 10.1038/43927 (doi:10.1038/43927) 10517640

[57] Martin-Castallanos C. Blanco M. A. de Prada J. M. Moreno S. 2000 The puc1 cyclin regulates the G1 phase of the fission yeast cell cycle in response to cell size. Mol. Biol. Cell 11, 543–554 10679013 10.1091/mbc.11.2.543 PMC14792

[58] Hagan I. Hayles J. Nurse P. 1988 Cloning and sequencing of the cyclin-related *cdc13*^+^ gene and a cytological study of its role in fission yeast mitosis. J. Cell Sci. 91, 587–595 2908246 10.1242/jcs.91.4.587

[59] Kozar K. 2004 Mouse development and cell proliferation in the absence of D-cyclins. Cell 118, 477–491 10.1016/j.cell.2004.07.025 (doi:10.1016/j.cell.2004.07.025) 15315760

[60] Geng Y. 2003 Cyclin E ablation in the mouse. Cell 114, 431–443 10.1016/S0092-8674(03)00645-7 (doi:10.1016/S0092-8674(03)00645-7) 12941272

[61] Liu D. Matzuk M. M. Sung W. K. Guo Q. Wang P. Wolgemuth D. J. 1998 Cyclin A1 is required for meiosis in the male mouse. Nat. Genet. 20, 377–380 10.1038/3855 (doi:10.1038/3855) 9843212

[62] Murphy M. Stinnakre M.-G. Senamaud-Beaufort C. Winston N. J. Sweeney C. Kubelka M. Carrington M. Brechot C. Sobczak-Thépot J. 1997 Delayed early embryonic lethality following disruption of the murine cyclin A2 gene. Nat. Genet. 15, 83–86 10.1038/ng0197-83 (doi:10.1038/ng0197-83) 8988174

[63] Kalaszczynska I. 2009 Cyclin A is redundant in fibroblasts but essential in hematopoietic and embryonic stem cells. Cell 138, 352–565 10.1016/j.cell.2009.04.062 (doi:10.1016/j.cell.2009.04.062) 19592082 PMC2745999

[64] Brandeis M. Rosewell I. Carrington M. Crompton T. Jacobs M. A. Kirk J. Gannon J. Hunt T. 1998 Cyclin B2-null mice develop normally and are fertile whereas cyclin B1-null mice die *in utero*. Proc. Natl Acad. Sci. USA 95, 4344–4349 10.1073/pnas.95.8.4344 (doi:10.1073/pnas.95.8.4344) 9539739 PMC22491

[65] Hochegger H. Takeda S. Hunt T. 2008 Cyclin-dependent kinases and cell-cycle transitions: does one fit all? Nat. Rev. Mol. Cell Biol. 9, 910–916 10.1038/nrm2510 (doi:10.1038/nrm2510) 18813291

[66] Lopez-Girona A. Mondesert O. Leatherwood J. Russell P. 1998 Negative regulation of Cdc18 DNA replication protein by Cdc2. Mol. Biol. Cell 9, 63–73 9436991 10.1091/mbc.9.1.63 PMC25219

[67] Merrick K. A. Larochelle S. Zhang C. Allen J. J. Shokat K. M. Fisher R. P. 2008 Distinct activation pathways confer cyclin-binding specificity on Cdk1 and Cdk2 in human cells. Mol. Cell 32, 662–672 10.1016/j.molcel.2008.10.022 (doi:10.1016/j.molcel.2008.10.022) 19061641 PMC2643088

[68] Santamaria D. 2007 Cdk1 is sufficient to drive the mammalian cell cycle. Nature 448, 811–815 10.1038/nature06046 (doi:10.1038/nature06046) 17700700

[69] Booher R. Beach D. 1988 Involvement of *cdc13*^*+*^ in mitosis control in *Schizosaccharomyces pombe*: possible interaction of the gene product with microtubules. EMBO J. 7, 2321–2327 2847913 10.1002/j.1460-2075.1988.tb03075.x PMC457096

[70] Forsburg S. L. Nurse P. 1991 Identification of a G1-type cyclin *puc1*^+^ in the fission yeast *Schizosaccharomyces pombe*. Nature 351, 245–248 10.1038/351245a0 (doi:10.1038/351245a0) 1828291

[71] Bueno A. Richardson H. Reed S. I. Russell P. 1991 A fission yeast B-type cyclin functioning early in the cell cycle. Cell 66, 149–159 10.1016/0092-8674(91)90147-Q (doi:10.1016/0092-8674(91)90147-Q) 1829983

[72] Bueno A. Russell P. 1993 Two fission yeast B-type cyclins, Cig2 and Cdc13, have different functions in mitosis. Mol. Cell. Biol. 13, 2286–2297 8455610 10.1128/mcb.13.4.2286-2297.1993 PMC359549

[73] Connolly T. Beach D. 1994 Interaction between the Cig1 and Cig2 B-type cyclins in the fission yeast cell cycle. Mol. Cell. Biol. 14, 768–776 8264644 10.1128/mcb.14.1.768-776.1994 PMC358425

[74] Obara-Ishihara T. Okayama H. 1994 A B-type cyclin negatively regulates conjugation via interacting with cell cycle ‘start’ genes in fission yeast. EMBO J. 13, 1863–1872 7909513 10.1002/j.1460-2075.1994.tb06455.x PMC395026

[75] Fisher D. L. Nurse P. 1996 A single fission yeast mitotic cyclin B p34^*cdc2*^ kinase promotes both S-phase and mitosis in the absence of G_1_ cyclins. EMBO J. 15, 850–860 8631306 PMC450283

[76] Moreno S. Nurse P. 1994 Regulation of progression through the G1 phase of the cell cycle by the *rum1*^+^ gene. Nature 367, 236–242 10.1038/367236a0 (doi:10.1038/367236a0) 8121488

[77] Stern B. Nurse P. 1998 Cyclin B proteolysis and the cyclin-dependent kinase inhibitor rum1p are required for pheromone-induced G_1_ arrest in fission yeast. Mol. Biol. Cell 9, 1309–1321 9614176 10.1091/mbc.9.6.1309 PMC25352

[78] Russell P. Nurse P. 1987 Negative regulation of mitosis by *wee1*^+^, a gene encoding a protein kinase homolog. Cell 49, 559–567 10.1016/0092-8674(87)90458-2 (doi:10.1016/0092-8674(87)90458-2) 3032459

[79] Gould K. L. Nurse P. 1989 Tyrosine phosphorylation of the fission yeast *cdc2*^+^ protein kinase regulates entry into mitosis. Nature 342, 39–45 10.1038/342039a0 (doi:10.1038/342039a0) 2682257

[80] Dunphy W. G. Newport J. W. 1989 Fission yeast p13 blocks mitotic activation and tyrosine dephosphorylation of the *Xenopus* *cdc2* protein kinase. Cell 58, 181–191 10.1016/0092-8674(89)90414-5 (doi:10.1016/0092-8674(89)90414-5) 2473838

[81] Morla A. O. Draetta G. Beach D. Wang J. Y. J. 1989 Reversible tyrosine phosphorylation of cdc2: dephosphorylation accompanies activation during entry into mitosis. Cell 58, 193–203 10.1016/0092-8674(89)90415-7 (doi:10.1016/0092-8674(89)90415-7) 2473839

[82] Ghaemmaghami S. Huh W.-K. Bower K. Howson R. W. Belle A. Dephoure N. O'Shea E. K. Weissman J. S. 2003 Global analysis of protein expression in yeast. Nature 425, 737–741 10.1038/nature02046 (doi:10.1038/nature02046) 14562106

[83] Glotzer M. Murray A. W. Kirschner M. W. 1991 Cyclin is degraded by the uniquitin pathway. Nature 349, 132–138 10.1038/349132a0 (doi:10.1038/349132a0) 1846030

[84] Wolf F. Wandke C. Isenberg N. Geley S. 2006 Dose-dependent effects of stable cyclin B1 on progression through mitosis in human cells. EMBO J. 25, 2802–2813 10.1038/sj.emboj.7601163 (doi:10.1038/sj.emboj.7601163) 16724106 PMC1500859

[85] Drapkin B. J. Lu Y. Procko A. L. Timney B. L. Cross F. R. 2009 Analysis of the mitotic exit control system using locked levels of stable mitotic cyclin. Mol. Syst. Biol. 5, 328 10.1038/msb.2009.78 (doi:10.1038/msb.2009.78) 19920813 PMC2795472

[86] Visintin R. Craig K. Hwang E. S. Prinz S. Tyers M. Amon A. 1998 The phosphatase Cdc14 triggers mitotic exit by reversal of Cdk-dependent phosphorylation. Mol. Cell 2, 709–718 10.1016/S1097-2765(00)80286-5 (doi:10.1016/S1097-2765(00)80286-5) 9885559

[87] Pereira G. Schiebel E. 2003 Separase regulates INCENP-Aurora B anaphase spindle function through Cdc14. Science 302, 2120–2124 10.1126/science.1091936 (doi:10.1126/science.1091936) 14605209

[88] Higuchi T. Uhlmann F. 2005 Stabilization of microtubule dynamics at anaphase onset promotes chromosome segregation. Nature 433, 171–176 10.1038/nature03240 (doi:10.1038/nature03240) 15650742 PMC2586334

[89] Zhu C. Lau E. Schwarzenbacher R. Bossy-Wetzel E. Jiang W. 2006 Spatiotemporal control of spindle midzone formation by PRC1 in human cells. Proc. Natl Acad. Sci. USA 103, 6196–6201 10.1073/pnas.0506926103 (doi:10.1073/pnas.0506926103) 16603632 PMC1458854

[90] Woodbury E. L. Morgan D. O. 2007 Cdk and APC activities limit the spindle-stabilizing function of Fin1 to anaphase. Nat. Cell Biol. 9, 106–112 10.1038/ncb1523 (doi:10.1038/ncb1523) 17173039

[91] Khmelinskii A. Roostalu J. Roque H. Antony C. Schiebel E. 2009 Phosphorylation-dependent protein interactions at the spindle midzone mediate cell cycle regulation of spindle elongation. Dev. Cell 17, 244–256 10.1016/j.devcel.2009.06.011 (doi:10.1016/j.devcel.2009.06.011) 19686685

[92] Stegmeier F. Amon A. 2004 Closing mitosis: the functions of the Cdc14 phosphatase and its regulation. Annu. Rev. Genet. 38, 203–231 10.1146/annurev.genet.38.072902.093051 (doi:10.1146/annurev.genet.38.072902.093051) 15568976

[93] Queralt E. Uhlmann F. 2008 Cdk-counteracting phosphatases unlock mitotic exit. Curr. Opin. Cell Biol. 20, 661–668 10.1016/j.ceb.2008.09.003 (doi:10.1016/j.ceb.2008.09.003) 18845253 PMC2605245

[94] Jin F. Liu H. Liang F. Rizkallah R. Hurt M. M. Wang Y. 2008 Temporal control of the dephosphorylation of Cdk substrates by mitotic exit pathways in budding yeast. Proc. Natl Acad. Sci. USA 105, 16 177–16 182 10.1073/pnas.0808719105 (doi:10.1073/pnas.0808719105) PMC2570984 18845678

[95] Stegmeier F. Visintin R. Amon A. 2002 Separase, polo kinase, the kinetochore protein Slk19, and Spo12 function in a network that controls Cdc14 localization during early anaphase. Cell 108, 207–220 10.1016/S0092-8674(02)00618-9 (doi:10.1016/S0092-8674(02)00618-9) 11832211

[96] Azzam R. Chen S. L. Shou W. Mah A. S. Alexandru G. Nasmyth K. Annan R. S. Carr S. A. Deshaies R. J. 2004 Phosphorylation by cyclin B-Cdk underlies release of mitotic exit activator Cdc14 from the nucleolus. Science 305, 516–519 10.1126/science.1099402 (doi:10.1126/science.1099402) 15273393

[97] Queralt E. Lehane C. Novak B. Uhlmann F. 2006 Downregulation of PP2A^Cdc55^ phosphatase by separase initiates mitotic exit in budding yeast. Cell 125, 719–732 10.1016/j.cell.2006.03.038 (doi:10.1016/j.cell.2006.03.038) 16713564

[98] Liakopoulos D. Kusch J. Grava S. Vogel J. Barral Y. 2003 Asymmetric loading of Kar9 onto spindle poles and microtubules ensures proper spindle alignment. Cell 112, 561–574 10.1016/S0092-8674(03)00119-3 (doi:10.1016/S0092-8674(03)00119-3) 12600318

[99] Mochida S. Ikeo S. Gannon J. Hunt T. 2009 Regulated activity of PP2A-B55d is crucial for controlling entry into and exit from mitosis in *Xenopus* egg extracts. EMBO J. 28, 2777–2785 10.1038/emboj.2009.238 (doi:10.1038/emboj.2009.238) 19696736 PMC2750019

[100] Wolfe B. A. McDonald W. H. Yates J. R. III Gould K. L. 2006 Phospho-regulation of the Cdc14/Clp1 phosphatase delays late mitotic events in *S. pombe*. Dev. Cell 11, 423–430 10.1016/j.devcel.2006.07.016 (doi:10.1016/j.devcel.2006.07.016) 16950131

[101] Bouchoux C. Uhlmann F. In press A quantitative model for ordered Cdk substrate dephosphorylation during mitotic exit. Cell (doi:10.1016/j.cell.2011.09.047) 10.1016/j.cell.2011.09.047 22078879

[102] Kinoshita N. Yamano H. Niwa H. Yoshida T. Yanagida M. 1993 Negative regulation of mitosis by the fission yeast protein phosphatase ppa2. Genes Dev. 7, 1059–1071 10.1101/gad.7.6.1059 (doi:10.1101/gad.7.6.1059) 8389306

[103] Wolfe B. A. Gould K. L. 2004 Fission yeast Clp1p phosphatase affects G_2_/M transition and mitotic exit through Cdc25p inactivation. EMBO J. 23, 919–929 10.1038/sj.emboj.7600103 (doi:10.1038/sj.emboj.7600103) 14765109 PMC381010

[104] Lee T. H. Turck C. Kirschner M. W. 1994 Inhibition of cdc2 activation by INH/PP2A. Mol. Biol. Cell 5, 323–338 8049524 10.1091/mbc.5.3.323 PMC301040

[105] Novak B. Tyson J. J. 1993 Numerical analysis of a comprehensive model of M-phase control in *Xenopus* oocyte extracts and intact embryos. J. Cell Sci. 106, 1153–1168 8126097 10.1242/jcs.106.4.1153

[106] Tyers M. Cook M. 2007 Size control goes global. Curr. Opin. Biotechnol. 18, 341–350 10.1016/j.copbio.2007.07.006 (doi:10.1016/j.copbio.2007.07.006) 17768045

[107] Nurse P. 2011 Obituary: Murdoch Mitchison 1922–2011. Nat. Cell Biol. 13, 520 10.1038/ncb0511-520 (doi:10.1038/ncb0511-520)

[108] Nurse P. 1975 Genetic control of cell size at cell division in yeast. Nature 256, 547–551 10.1038/256547a0 (doi:10.1038/256547a0) 1165770

[109] Martin S. G. Berthelot-Grosjean M. 2009 Polar gradients of the DYRK-family kinase Pom1 couple cell length with the cell cycle. Nature 459, 852–856 10.1038/nature08054 (doi:10.1038/nature08054) 19474792

[110] Moseley J. B. Mayeux A. Paoletti A. Nurse P. 2009 A spatial gradient coordinates cell size and mitotic entry in fission yeast. Nature 459, 857–860 10.1038/nature08074 (doi:10.1038/nature08074) 19474789

[111] Jorgensen P. Nishikawa J. L. Breitkreutz B.-J. Tyers M. 2002 Systematic identification of pathways that couple cell growth and division in yeast. Science 297, 395–400 10.1126/science.1070850 (doi:10.1126/science.1070850) 12089449

[112] Zhurinsky J. Leonhard K. Watt S. Marguerat S. Bähler J. Nurse P. 2010 A coordinated global control over cellular transcription. Curr. Biol. 20, 2010–2015 10.1016/j.cub.2010.10.002 (doi:10.1016/j.cub.2010.10.002) 20970341

[113] Tokiwa G. Tyers M. Vople T. Futcher B. 1994 Inhibition of G1 cyclin activity by the Ras/cAMP pathway in yeast. Nature 371, 342–345 10.1038/371342a0 (doi:10.1038/371342a0) 8090204

[114] Wang H. Carey L. B. Cai Y. Wijnen H. Futcher B. 2009 Recruitment of Cln3 cyclin to promoters controls cell cycle entry via histone deacetylase and other targets. PLoS Biol. 7, e1000189 10.1371/journal.pbio.1000189 (doi:10.1371/journal.pbio.1000189) 19823669 PMC2730028

